# Increased brain age in adults with Prader-Willi syndrome

**DOI:** 10.1016/j.nicl.2019.101664

**Published:** 2019-01-10

**Authors:** Adriana M. Azor, James H. Cole, Anthony J. Holland, Maureen Dumba, Maneesh C. Patel, Angelique Sadlon, Anthony P. Goldstone, Katherine E. Manning

**Affiliations:** a Computational, Cognitive and Clinical Neuroimaging Laboratory, Division of Brain Sciences, Imperial College London, Hammersmith Hospital, London, UK; b Cambridge Intellectual and Developmental Disabilities Research Group, Academic Department of Psychiatry, University of Cambridge, Cambridge, UK; c National Institute for Health Research (NIHR) Collaborations for Leadership in Applied Health Care Research and Care (CLAHRC), East of England, UK; d Department of Radiology, Imperial College Healthcare NHS Trust, London, UK; e PsychoNeuroEndocrinology Research Group, Neuropsychopharmacology Unit, Centre for Psychiatry, Division of Brain Sciences, Imperial College London, Hammersmith Hospital, London, UK

**Keywords:** Body mass index, MRI, Structural neuroimaging, SNORD116, PWS, Obesity, BMI, body mass index, GM, grey matter, PAD, predicted-age difference, PWS, Prader-Willi syndrome, WM, white matter

## Abstract

Prader-Willi syndrome (PWS) is the most common genetic obesity syndrome, with associated learning difficulties, neuroendocrine deficits, and behavioural and psychiatric problems. As the life expectancy of individuals with PWS increases, there is concern that alterations in brain structure associated with the syndrome, as a direct result of absent expression of PWS genes, and its metabolic complications and hormonal deficits, might cause early onset of physiological and brain aging.

In this study, a machine learning approach was used to predict brain age based on grey matter (GM) and white matter (WM) maps derived from structural neuroimaging data using T1-weighted magnetic resonance imaging (MRI) scans. Brain-predicted age difference (brain-PAD) scores, calculated as the difference between chronological age and brain-predicted age, are designed to reflect deviations from healthy brain aging, with higher brain-PAD scores indicating premature aging.

Two separate adult cohorts underwent brain-predicted age calculation. The main cohort consisted of adults with PWS (*n* = 20; age mean 23.1 years, range 19.8–27.7; 70.0% male; body mass index (BMI) mean 30.1 kg/m^2^, 21.5–47.7; *n* = 19 paternal chromosome 15q11–13 deletion) and age- and sex-matched controls (*n* = 40; age 22.9 years, 19.6–29.0; 65.0% male; BMI 24.1 kg/m^2^, 19.2–34.2) adults (BMI PWS vs. control *P* = .002). Brain-PAD was significantly greater in PWS than controls (effect size mean ± SEM +7.24 ± 2.20 years [95% CI 2.83, 11.63], P = .002). Brain-PAD remained significantly greater in PWS than controls when restricting analysis to a sub-cohort matched for BMI consisting of *n* = 15 with PWS with BMI range 21.5–33.7 kg/m^2^, and *n* = 29 controls with BMI 21.7–34.2 kg/m^2^ (effect size +5.51 ± 2.56 years [95% CI 3.44, 10.38], *P* = .037). In the PWS group, brain-PAD scores were not associated with intelligence quotient (IQ), use of hormonal and psychotropic medications, nor severity of repetitive or disruptive behaviours. A 24.5 year old man (BMI 36.9 kg/m^2^) with PWS from a SNORD116 microdeletion also had increased brain PAD of 12.87 years, compared to 0.84 ± 6.52 years in a second control adult cohort (*n* = 95; age mean 34.0 years, range 19.9–55.5; 38.9% male; BMI 28.7 kg/m^2^, 19.1–43.1).

This increase in brain-PAD in adults with PWS indicates abnormal brain structure that may reflect premature brain aging or abnormal brain development. The similar finding in a rare patient with a SNORD116 microdeletion implicates a potential causative role for this PWS region gene cluster in the structural brain abnormalities associated primarily with the syndrome and/or its complications. Further longitudinal neuroimaging studies are needed to clarify the natural history of this increase in brain age in PWS, its relationship with obesity, and whether similar findings are seen in those with PWS from maternal uniparental disomy.

## Highlights

• Prader-Willi syndrome is the commonest genetic obesity syndrome.
• 19 of the 20 adults with PWS had a paternal deletion in chromosome 15q11–13.
• Adults with PWS had increased brain age using structural MRI with machine learning.
• This was independent of their higher BMI, and use of growth and sex hormones.
• Another man with a SNORD116 microdeletion also had an increased brain age.

## 1 Introduction

Prader-Willi Syndrome (PWS) is a multi-system, genetically determined, neurodevelopmental disorder arising from the loss of expression of genes of paternal inheritance on chromosome 15 (Bittel and Butler, 2005). PWS results from a paternal deletion of chromosome 15q11-q13 in 75% of cases (Amos-Landgraf et al., 1999), maternal uniparental disomy (mUPD) in 24%, and genetic imprinting errors in 1% of cases (Horsthemke and Buiting, 2006; Goldstone et al., 2008).

PWS has an estimated birth incidence of 1 in 27,000 individuals and presents with a characteristic but variable phenotype affecting many organ systems (Whittington et al., 2001; Gunay-Aygun et al., 2001; Smith et al., 2003). PWS is characterised by its nutritional phases from initial feeding difficulties and failure-to-thrive in infancy, to progressive hyperphagia and morbid obesity (if access to food is not controlled) from later childhood to adulthood, growth hormone (GH) and sex hormone deficiencies, secondary to hypothalamic dysfunction (Miller et al., 2007; Goldstone et al., 2008; Goldstone et al., 2012), and possible pro-hormone/−neuropeptide precursor processing defects (Burnett et al., 2017). PWS is also associated with developmental delay, mild to moderate intellectual disability, and behavioural and psychiatric disturbances (Cassidy and Driscoll, 2009).

In the past, mortality rates for people with intellectual disabilities was higher than it is now (Maaskant et al., 2002), and in recent years, the life expectancy of people with PWS has increased, thanks to improvements in clinical management (Whittington et al., 2015). However, increased morbidity and secondary complications, such as respiratory infections and cardiorespiratory failure related to obesity, continue to be the major cause of death in individuals with PWS (Schrander-Stumpel et al., 2004; Vogels et al., 2004). Reports of older people with PWS are scarce, as most research has focused on clinical characteristics of PWS in childhood, adolescence and early adulthood (Sinnema et al., 2012).

People with PWS have abnormal development in certain brain areas (Manning and Holland, 2015), with some structural differences depending on their genetic subtypes and varying between early atrophy and arrested development (Lukoshe et al., 2013). Two main competing hypotheses may explain the neurological and behavioural changes associated with PWS:

1. *Premature aging and brain atrophy*. Reports from older PWS patients describe cases of cortical atrophy and signs of premature aging, similar to that seen in other neurodegenerative diseases (Sinnema et al., 2012), cognitive and functional decline affecting language, memory, orientation, and behaviour, with higher incidence in women (Maaskant et al., 2002; Whittington et al., 2015;), and cases meeting criteria of Alzheimer-type dementia (Sinnema et al., 2010; Whittington et al., 2015).

2. *Atypical and arrested development*. Arrested development in PWS might explain some core phenotypes such as repetitive behaviour and tantrums (Holland et al., 2003). Some neuroimaging findings also indicate a fundamental arrest in brain development in children with PWS, or a divergence from the normal developmental pattern (Lukoshe et al., 2013).

Given the uncertainties of brain development and aging in PWS, a better understanding of neurological abnormalities and age-related changes in the brain in comparison to typical development is needed to allow better prediction of health outcomes and to provide insights into the role of parental-origin specific genomic imprinting from a developmental and evolutionary point of view.

Study of rare mutations or microdeletions of specific PWS genes can also reveal much about genotype-phenotype associations in PWS. Recent rare case reports have revealed that smaller deletions of some genes on chromosome 15q11–13 can lead to features associated with PWS, even in the absence of the full diagnostic criteria for PWS, including a ~187 kb microdeletion encompassing several small nucleolar RNAs (snoRNAs) gene clusters, especially the SNORD116 cluster. The phenotype of such cases includes hyperphagia, severe obesity, learning difficulties and hypogonadism (Sahoo et al., 2008; de Smith et al., 2009; Duker et al., 2010; Bieth et al., 2015).

To evaluate age-related changes to brain structure, neuroimaging data has been used to predict chronological age based on a multivariate machine-learning model of healthy individuals (Franke et al., 2010; Cole et al., 2018). Using this paradigm, both genetic and environmental factors have been reported to influence brain aging. These include obesity and type 2 diabetes mellitus (T2DM) (Franke et al., 2013; Ronan et al., 2016), HIV disease (Cole et al., 2017b), and certain neuropsychiatric disorders (Gaser et al., 2013; Koutsouleris et al., 2014; Schnack et al., 2016), which cause the brain to appear older than it actually is chronologically. Meanwhile, neuroprotective factors, such as physical exercise and higher cognitive reserve, have been associated with slower brain aging (Luders et al., 2016; Steffener et al., 2016). Recently, this model was used to show that genetic Down syndrome (trisomy of chromosome 21) is associated with early onset of structural brain changes corresponding to premature brain aging (Cole et al., 2017a). The deviation from the normal trend of brain maturation and aging seems to be an accurate indication of important neurological changes associated with physiological abnormalities in the elderly, and indeed is a predictor of mortality (Cole et al., 2018).

In this paper, we investigated brain age in young adults with PWS compared to healthy individuals, to determine whether they exhibited patterns of arrested development or premature aging. We also explored whether brain structural changes were related to common clinical characteristics of PWS, namely high body mass index (BMI), low intellectual quotient (IQ), hormonal status, psychiatric medication use, and behavioural measures, to identify possible mechanisms and associations with the abnormalities accompanying PWS. We also examined brain age in one of the rare cases of PWS due to a SNORD116 microdeletion to identify potential gene(s) in the PWS chromosomal region contributing to changes in brain age (de Smith et al., 2009).

## 2 Subjects and methods

### 2.1 Participants

#### 2.1.1 Training set

Training sets are typically used in machine learning to discover relationship patterns that can be applied for future predictions without explicit input by the researcher. In this case, T1-weighted MRI scans were collected from public sources (Supplementary Table 1), and included 2001 typically developing individuals (1016 males (50.8%), age mean ± SD 37.0 ± 18.1 years, range 18.0–90.0). The participants were screened according to their respective study's criteria, to ensure the absence of psychological or neurological diagnosis, and major health conditions. The scans were used to derive a statistical model of healthy brain structure across the lifespan.

#### 2.1.2 Test sets

The model constructed by the training set was then applied for predictive analysis on the test set. In our study, the test sets comprised two separate adult cohorts.

##### 2.1.2.1 Cohort 1 - PWS and matched controls

This cohort included *n* = 20 adults with PWS (mean ± SD age 23.1 ± 2.4 years) and *n* = 40 age- and sex-matched, typically developing controls (age 22.9 ± 2.2 years) (Table 1). All participants were recruited at the University of Cambridge to undergo MRI scans. Participants needed to be between 18 and 28 years old, of either sex and have the capacity to consent. Written informed consent was given by all participants. All participants had height and weight recorded. This cohort has previously been described in (Manning and Holland, 2015). Typically developing control participants aged 18–24 years (*n* = 36) were recruited and tested as part of the NeuroScience in Psychiatry Network (NSPN) U-Change project, enabling the selection of age and sex-matched controls. Additional control participants aged 24–28 years (*n* = 4) were recruited from the local population.

**Table 1:** Characteristics of Prader-Willi syndrome and controls (cohort 1).

	PWS	Control	*P*-value^a^
n	20	40	
Demographic and anthropometric data			
Age (y)	22.6 [21.3, 24.8] (19–27)	22.4 [21.3, 24.3] (19–29)	0.74
Male n (%)	14 (70.0%)	26 (65.0%)	0.70
BMI (kg/m^2^)	30.1 ± 7.2 (21.5–47.7)	24.1 ± 3.8 (19.2–34.2)	<0.001
T2DM n (%)	4 (20.0%)	NA	NA
Genotype (n)	19 Del, 1 mUPD	NA	NA

Psychological measurements			
IQ	63.1 ± 11.9 (48–95)	112.9 ± 11.2 (81–132)	<0.001
DBC-A disruptive (max 48)	17.6 ± 9.9 (2–43)	NA	NA
RBS-R - sameness (max 33)	6.8 ± 7.0 (1–24)	NA	NA

Medications			
Growth hormone n (%)	Ever 17 (85.0%)	0	NA
	Current 7 (35.0%)		
Sex hormones n (%)	Ever 15 (75.0%)	0	NA
	Current 7 (35.0%)		
Psychoactive n (%)	10 (50.0%)^b^	0	NA
Anti-psychotic n (%)	3 (15.0%)^b^	0	NA
Anti-depressants n (%)	7 (35.0%)^b^	0	NA
Benzodiazepines n (%)	1 (5.0%)^b^	0	NA
Anti-convulsant n (%)	1 (5.0%)^b^	0	NA
Anti-cholinergic n (%)	1 (5.0%)^b^	0	NA
Diabetes n (%)	3 (15%)^b^	0	NA

All data given as mean ± SD (range), median [interquartile range] (range), or n (%).

Abbreviations: DBC-A: disruptive behaviour scale, Del: deletion, mUPD: maternal uniparental disomy, NA: not applicable, RBS: repetitive behaviour scale, T2DM: type 2 diabetes mellitus.

a *P* value for two-way unpaired t-test, Mann-Whitney U test, or chi-squared test.

b currently receiving; note that in the PWS group, one participant was taking both an anti-depressant and an anti-psychotic; another was taking both an anti-depressant and a benzodiazepine; and another was taking both an anti-psychotic and an anti-cholinergic.

For the PWS group, where genetic diagnosis of subtypes was not available in medical records, the genotype was confirmed using blood or saliva samples. Records of medication and hormonal therapies were given by participants, parents and care-givers. IQ was assessed in the PWS group using the Wechsler Adult Intelligence Scale Fourth Edition (WAIS-IV; Pearson, London, UK). For the control group, a shortened version of the WAIS was used (vocabulary and matrix reasoning subtests) from the Wechsler Abbreviated Scale of Intelligence Second Edition (WASI-II; Pearson, London, UK). The Developmental Behaviour Checklist for Adults (DBC-A) was used to assess behaviour difficulties and temper outbursts in PWS using DBC-A disruptive score (Mohr et al., 2005). The Repetitive Behaviour Scale - Revised (RBS-R) was used to measure the severity of repetitive behaviours using RBS-R sameness score (Bodfish et al., 2000).

Exclusion criteria were: inability to tolerate MRI scans, history of psychiatric disorder for controls or current psychiatric disorder disrupting compliance with study demands for PWS participants, history of neurological disease or trauma, currently or recently participating in a clinical trial for medicinal investigation, metal object precluding MRI, and currently being treated for drug or alcohol dependency. Ethical approval for the study was granted by the Cambridge East (East of England) NHS National Research Ethics Committee (REC number 13/EE/0373).

##### 2.1.2.2 Cohort 2 - SNORD116 microdeletion and controls

One adult male of Indian ethnicity with a SNORD116 microdeletion, previously reported in (de Smith et al., 2009), was recruited from the PWS and genetic obesity clinic at Hammersmith Hospital, Imperial College Healthcare NHS trust, London, run by A.P.G., with written informed consent, for an MRI brain scan (West London 1 REC, number 10/H0707/60). At the time of imaging he was 24.5 years old with BMI 36.9 kg/m^2^ (Table 2).

**Table 2:** Characteristics of SNORD116 microdeletion patient and controls (cohort 2).

	SNORD116 microdeletion	Control
n	1	95
Demographic and anthropometric data		
Age (y)	24.5	34.0 ± 10.2 (19.9–55.5)
Male n (%)	1	37 (34.0%)
BMI (kg/m^2^)	36.9	28.7 ± 6.6 (19.1–53.1)
T2DM n (%)	1	3 (3.2%)

Medications		
Growth hormone n (%)	0	0
Sex hormones n (%)	1^a^	0
Anti-psychotic n (%)	1^a^	0
Anti-convulsant n (%)	1^a^	0
Diabetes n (%)	1^a^	3 (3.2%)^a^
Lipid lowering medication n (%)	0	2 (2.1%)^a^
Anti-hypertensives n (%)	0	4 (4.2%)^a^

All data given as mean ± SD (range) or n (%). Abbreviations: BP: blood pressure, T2DM: type 2 diabetes mellitus.

a Currently receiving.

A total of *n* = 95 adults were included in the control group for the patient with the SNORD116 microdeletion, consisting of *n* = 30 with BMI <25.0 kg/m^2^, *n* = 37 with BMI ≥25.0 and < 30.0 kg/m^2^, and *n* = 28 with BMI ≥30.0 (Table 2). All controls had previously taken part in one of six studies investigating the effects of hormones, obesity surgery and dietary manipulations on brain activation in functional MRI studies (REC 07/Q0406/19, 08/Q0707/139, 08/H0707/99, 09/H0709/18, 09/H0707/30, 10/H0707/60). All subjects gave written informed consent. Inclusion criteria were BMI between 18.0 and 50.0 kg/m^2^, age 18–60 years of either sex. Exclusion criteria were claustrophobia, metal object precluding MRI, inability to read and understand English, T2DM receiving insulin or GLP-1 receptor agonist therapy, type 1 diabetes mellitus, current smoker, history of cancer, epilepsy, cerebrovascular accident, ischaemic heart disease, cardiac arrhythmia, respiratory disease other than mild asthma, alcohol or other drug dependence, significant renal, gastrointestinal, hepatic disease, or endocrine disease other than controlled and treated primary hypothyroidism, known prematurity (gestation <32 weeks), unstable depression (dose change of anti-depressants in last 3 months). Subjects on anti-hypertensive, lipid lowering medication or anti-depressant medication were not excluded.

### 2.2 Procedure

#### 2.2.1 Neuroimaging data acquisition

For the training set, the acquisition of three-dimensional T1-weighted MRI scans was conducted using various parameters and field strengths (either 1.5T or 3T) depending on local study protocols (Supplementary Table 1).

##### 2.2.1.1 PWS and matched controls

Three-dimensional, high-resolution, T1-weighted MRI scans for the acquisition of structural images for the PWS and control groups, used a Siemens 3T scanner (Siemens MAGNETON 3T Trio), at the Wolfson Brain Imaging Centre, University of Cambridge, Addenbrooke's Hospital, UK, using a 32-channel head coil, with the following acquisition parameters: echo time (TE) 2.20 ms, repetition time (TR) 18.70 ms, flip angle 20°, field-of-view 256 mm, voxel dimensions 1.0 × 1.0 × 1.0 mm.

##### 2.2.1.2 SNORD116 microdeletion and controls

Three-dimensional, high-resolution, T1-weighted turbo field echo brain scans collected from 3T Philips Achieva scanner, Robert Steiner MRI Unit, MRC Clinical Sciences Centre, Imperial College London, UK, using an 8-channel head coil with the following acquisition parameters: echo time (TE) 4.6 ms, repetition time (TR) 9.7 ms, flip angle 8°, field-of-view 240 mm, voxel dimensions 0.94 × 0.94 × 1.2 mm.

#### 2.2.2 Neuroradiological reports

Two independent neuroradiologists reviewed all of the T1 isotropic images in cohort 1 from 20 patients with PWS and 40 controls (M.D. Neuroradiology Fellow; M.P. Consultant Neuroradiologist). The patient with a SNORD116 microdeletion additionally had T2, FLAIR and dedicated MRI pituitary images (without gadolinium contrast) reviewed. Both were blinded to the identity of the patients. All abnormalities were documented, including previously described intracranial defects in PWS such as ventriculomegaly, peri-sylvian polymicrogyria, abnormal insula closure and parieto-occipital abnormalities (Manning and Holland, 2015).

The T1 MRI brain scans of the *n* = 95 controls in cohort 2 (for the SNORD116 microdeletion patient) had previously been reported by a Consultant Neuroradiologist as having no clinically significant abnormalities as part of routine clinical governance for our neuroimaging research studies.

#### 2.2.3 Brain-predicted age calculation

**Fig. 1:**
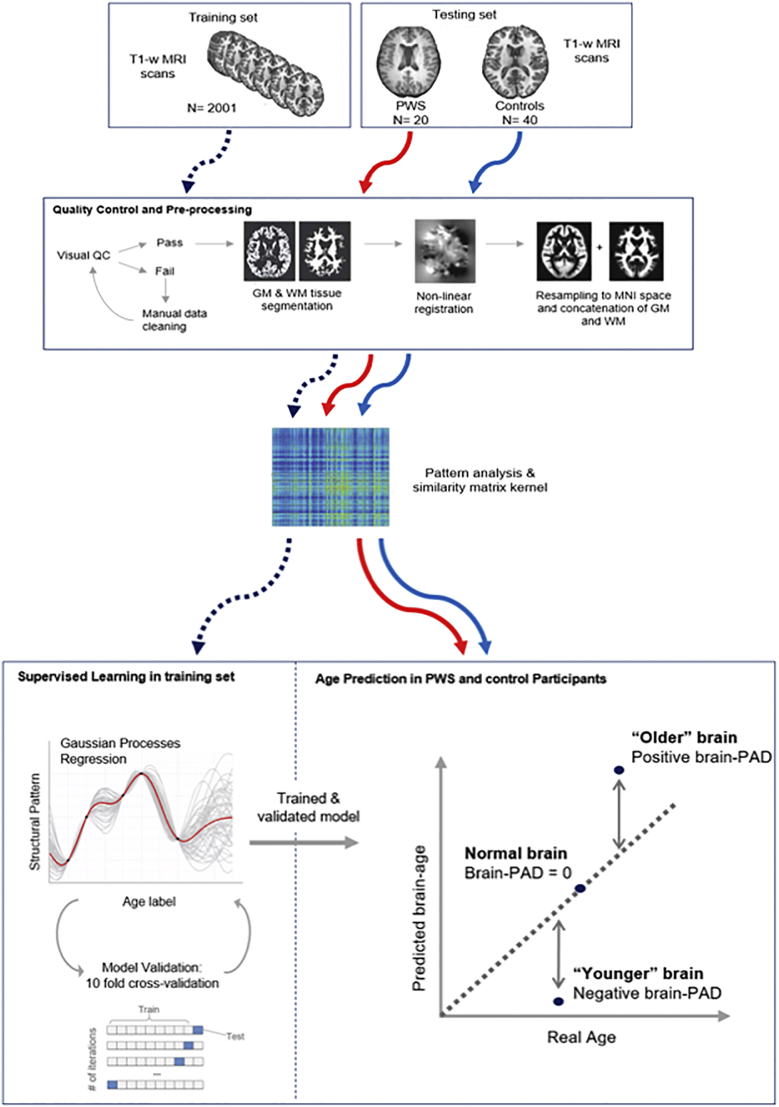
Outline of methods for brain-age prediction using machine learning. The data includes a training set and 2 testing sets (cohort1: controls vs. PWS; cohort 2: controls vs. SNORD116 microdeletion). The data was pre-processed using the Statistical Parametric Mapping software (SPM). The T1 images underwent tissue segmentation after initial quality control, separating GM, WM and CSF. The segmented images were then normalized using DARTEL for nonlinear registration, then resampled to the MNI152 template using a 4 mm smoothing kernel. GM and WM maps were concatenated into a single vector of data relating to brain size. All data underwent voxel-wise similarity analysis to generate a similarity kernel using PRoNTo. The training set was used to generate the brain-age model via supervised machine learning that produced a Gaussian Processes Regression (GPR) model trained to recognize structural patterns from imaging data associated with chronological age. The accuracy of the model generated from the training set was assessed using a 10-fold cross-validation method whereby 10% of samples were used for testing in all possible iterations to generate age predictions on all samples. The trained and validated GPR model was applied to the two test groups.

An overview of the methods is represented in Fig. 1. The protocol for the brain-age prediction procedure was previously outlined in (Cole et al., 2017b).

Acquired MRI data were preprocessed using the Statistical Parametric Mapping (SPM12) software (University College London, UK). The T1-weighted images underwent bias correction before being segmented into grey matter (GM), white matter (WM) and cerebral spinal fluid (CSF). Intracranial volume (ICV) was calculated as the sum of GM, WM and CSF. The segmentation process was followed by a quality check to verify the precision of the segmentation. At this stage, images from three PWS participants had to be re-segmented, as they failed the initial SPM segmentation. This was done using the FMRIB Software Library Brain Extraction Tool (FSL BET) to remove extraneous non-brain tissue from the field-of-view, after manual realignment of the head images and resetting of the origin to the anterior commissure in SPM. Preprocessing of the T1-weighted images ensured alignment and corresponding voxel-wise analysis when running the machine learning analysis. The resulting 3D maps were registered to a group-averaged template in Montreal Neurological Institute (MNI152) using the nonlinear SPM-DARTEL algorithm and smoothed with a 4 mm kernel.

Age predictions were based on the analysis of the normalized GM and WM maps, and were generated using the Pattern Recognition for Neuroimaging Toolbox (PRoNTo v2.0) (Schrouff et al., 2013). For each individual, the spatially normalized maps of GM and WM were transformed into vectors which were then concatenated together for each participant. An N x N similarity matrix was obtained by calculating the dot product of image vectors for every pair of participants. The machine learning process used a Gaussian Processes Regression (GPR) model, trained to predict chronological age based on structural patterns in brain imaging data in the healthy training set (*n* = 2001) (Cole et al., 2015).

To validate the age prediction model, a 10-fold cross-validation was applied to the training set, whereby the data is randomly split into 90% for training the model and 10% for testing the model. This procedure was repeated so that all data served as both training and testing. The accuracy of the model was calculated as the correlation between the real age and the predicted age, and the statistical significance of the model was computed using a permutation test (x1000).

Once validated, the model was applied on the two testing populations (PWS and controls from cohort 1, SNORD116 microdeletion and controls from cohort 2), 3D maps of GM and WM, to estimate the age of the participants based on their brain structure. Brain predicted-age difference (brain-PAD) scores were then calculated for each participant by subtracting the real age from the predicted age. Accordingly, a positive brain-PAD value would indicate an older-appearing brain.

### 2.3 Statistical analysis

Statistical analysis was conducted using R v3.3.3 (www.R-project.org) and SPSS v24, to assess normality using Kolmogorov–Smirnov test, relationships between variables and compare experimental groups. For brain-age prediction, brain-PAD scores were set as the dependent variable, and co-variates for linear regression analysis included BMI, IQ, WM and GM volumes, ICV, or behavioural measures, to study the effect of a variable on brain-PAD within groups and between groups (PWS and controls), reporting Pearson's correlation coefficients. Brain-PAD scores were also regressed against chronological real age. Comparison between PWS and controls, and within groups between sexes and medication or hormone use, used two-way unpaired *t*-test, or Mann-Whitney *U* test for data that was not normally distributed.

## 3 Results

### 3.1 Cohort description

#### 3.1.1 Cohort 1 - PWS and matched controls

Demographics of the PWS and matched control cohort are given in Table 1. The mean age of the two groups (PWS and controls) and the ratio of males to females in both groups were similar (*P* = .73 and *P* = .70 respectively). The mean IQ was significantly lower in the PWS group (effect size mean ± SEM 49.8 ± 3.1 [95% CI 43.5, 56.1], *t* = −15.9, *P* < .001), and the mean BMI was significantly higher in the PWS group (effect size 6.0 ± 1.4 [3.2, 8.8], *t* = −4.26, P < .001). Of the 20 participants with PWS, 17 had been treated with growth hormone (7 of whom were still on GH at the time of the MRI scan), and 15 had been treated with sex hormones (7 of whom were still receiving these). One of the PWS subjects had maternal UPD, while the remaining 19 had a paternal chromosome 15q11–13 deletion.

#### 3.1.2 Cohort 2 - SNORD116 microdeletion and controls

Demographics of the man with SNORD116 microdeletion and his control comparison group are given in Table 2. He had never received GH replacement but had received testosterone replacement from age 18 years. He was taking metformin for T2DM, and an anti-psychotic medication (Quetiapine) and anti-convulsant medication (Carbamazepine) for mood stabilization. In the control group, 3 participants were on medications for T2DM, 2 were on lipid lowering therapy, and 4 were on anti-hypertensive medications (Table 2).

### 3.2 Neuroradiological findings in PWS cohorts

In cohort 1, one out of the 20 PWS participants had dilated ventricles and widened sulci, and one arrested hydrocephalus with possible aqueduct stenosis (in total 10.0% of the PWS scans having clinically significant abnormalities), but none of these abnormalities were seen in the controls (Table 3). Peri-sylvian polymicrogyria, abnormal insula closure and parieto-occipital abnormalities were not seen in any subjects with PWS.

**Table 3:** Summary of radiological reports from T1-weighted MRI scans of PWS and control participants (cohort 1).

Group	n (%)	Findings
PWS (n = 20)	18 (90.0%)	Normal scan
	1 (5.0%)	Dilated ventricles and widened sulci
	1 (5.0%)	Arrested hydrocephalus, possible aqueduct stenosis
Control (n = 40)	34 (85.0%)	Normal scan
	2 (5.0%)	Developmental venous anomaly
	4 (10.0%)	Other benign findings^a^

a Other findings include small low signal lesion in right genu (possible incidental neuroepithelial cyst), maxillary sinus mucus, mega cisterna magna, small lipoma/dermoid in infundibular recess. None of the findings met exclusion criteria.

The man with a SNORD116 microdeletion had no visible abnormality on T1-, T2-weighted or FLAIR MRI brain scans or dedicated pituitary MRI scans.

### 3.3 Prediction of age using neuroimaging data is accurate

The chronological age of the training set was accurately predicted by the machine learning model using the 3D T1-weighted MRI scans (Cole et al., 2017b). The ten-fold cross-validation showed a correlation of r = +0.94 (significant after 1000 permutations correction, *P* < .01) between brain-predicted age and chronological age and explained 88% of the variance (R^2^). The mean absolute error (MAE) and root mean squared error (RMSE) of prediction were respectively 5.01 and 6.31 years. This stage validated our machine learning model as a predictor of age based on neuroimaging data for use on our test set consisting of controls and PWS participants.

### 3.4 Individuals with PWS have higher brain-predicted age difference

**Fig. 2:**
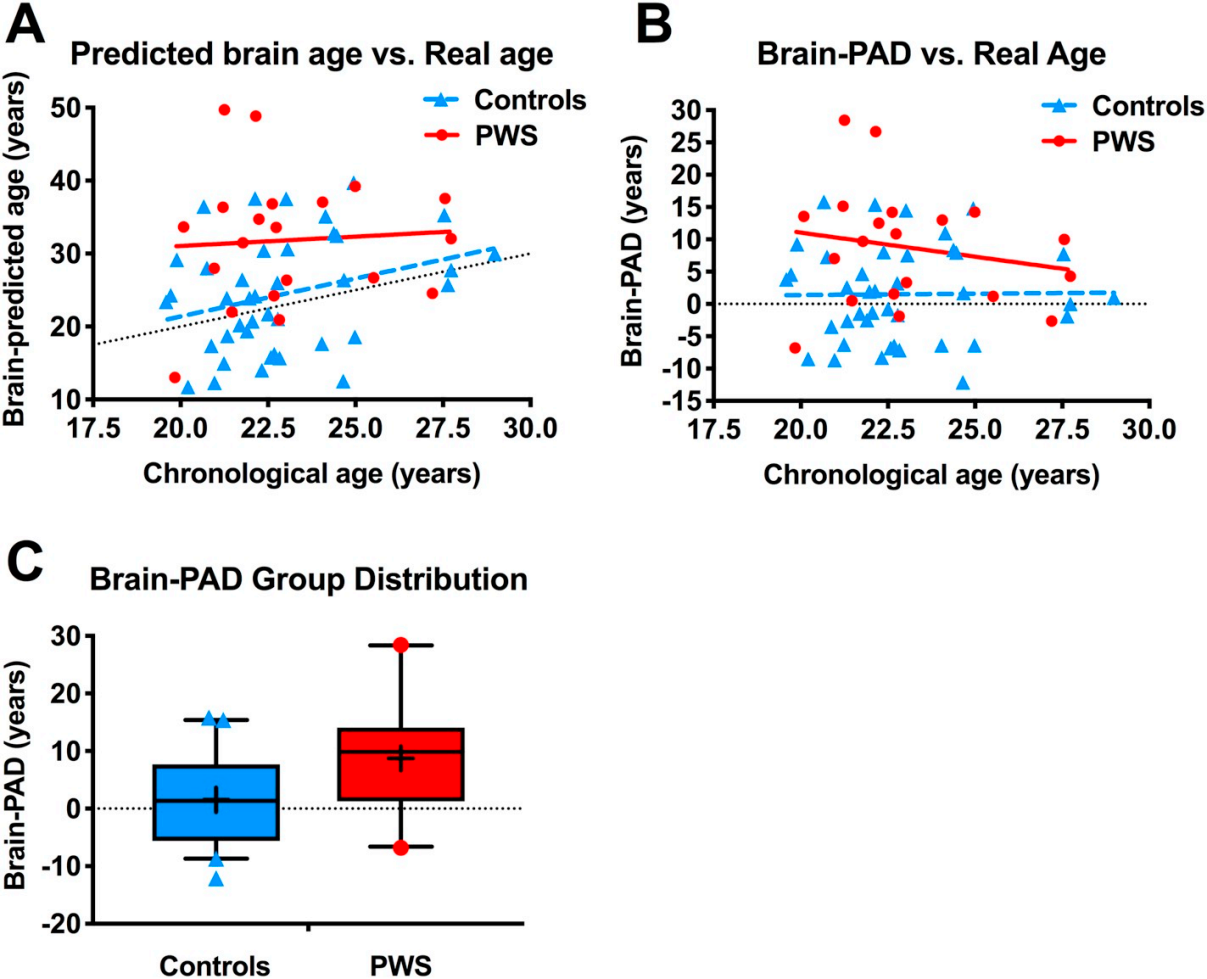
Brain-predicted age in individuals with Prader-Willi syndrome (PWS) and controls (cohort 1). (A,B) Scatterplot of (A) brain-predicted age or (B) brain-predicted age difference (brain-PAD) (y-axis) vs. chronological real age (x-axis) in controls (blue triangles, blue dashed linear regression line) and individuals with PWS (red circles, red solid linear regression line), with line of identity (black dotted line). (C) Boxplot shows brain-PAD scores distribution with median, interquartile range, and bars showing 5th and 95th percentiles, with outliers as symbols, and mean as a cross. (For interpretation of the references to color in this figure legend, the reader is referred to the web version of this article.)

Participants with PWS showed significantly higher brain-PAD scores (mean ± SD +8.74 ± 9.14 years) than the control group (+1.50 ± 7.42 years), with overall effect size mean ± SEM 7.24 ± 2.20 years [95% CI 2.83, 11.63], *t* = 3.29, *P* = .002 (Fig. 2).

Two participants with PWS showed very high brain-PAD scores (> + 25 years). However, brain-PAD scores remained greater in PWS than controls when excluding these subjects (effect size mean ± SEM +5.14 ± 2.06 years [95% CI 1.02, 9.27], *t* = 2.50, *P* = .015), so these potential outliers were not driving the main group effect.

### 3.5 Relationship of brain-PAD with GM and WM volumes

There was no significant difference in ICV or other tissue volumes between PWS and control groups (Table 4), and no significant overall effect of ICV on brain-PAD when controlling for group differences (β = −8.48 years [95% CI -25.00, 8.05], SE 8.25, *P* = .31, adjusted r^2^ = 0.17).

**Table 4:** Brain tissue volumes in PWS and control groups (cohort 1).

	PWS	Control	P-value
WM volume (cm^3^)	0.480 ± 0.083	0.478 ± 0.070	0.92
GM volume (cm^3^)	0.662 ± 0.106	0.703 ± 0.104	0.16
CSF volume (cm^3^)	0.233 [0.198, 0.278]	0.238 [0.187, 0.317]	0.82^a^
ICV (cm^3^)	1.396 [1.308, 1.473]	1.430 [1.312, 1.545]	0.16^a^

Data given as mean ± SD, or median [interquartile range], in cm^3^ for white matter (WM), grey matter (GM), cerebrospinal fluid (CSF) volumes and total intracranial volume (ICV), with *P*-values for differences in between groups by 2-way unpaired t-test or ^a^ Mann-Whitney U test.

**Fig. 3:**
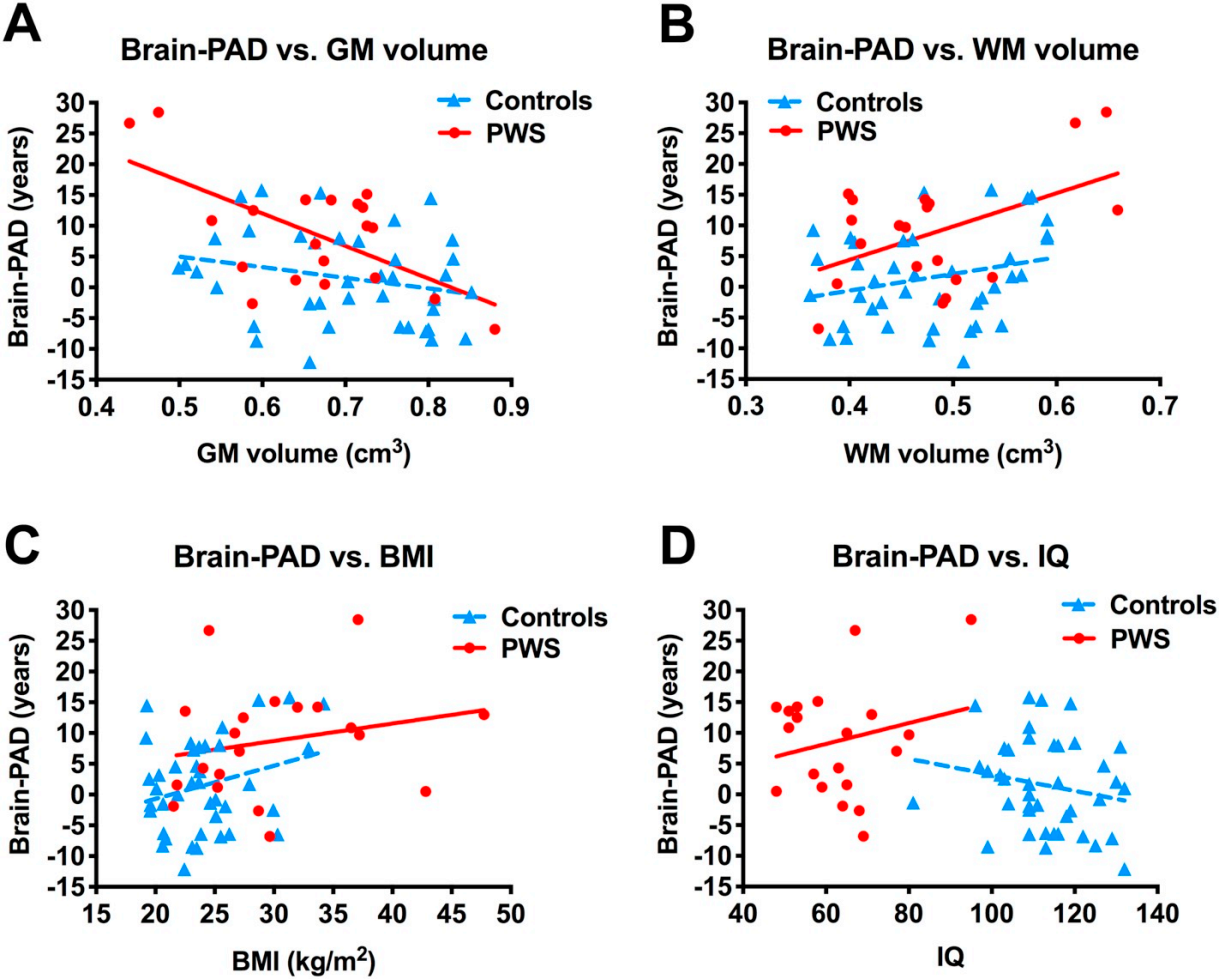
Brain-predicted age difference in individuals with Prader-Willi syndrome (PWS) and controls (cohort 1). Scatterplot of brain-predicted age difference (brain-PAD) (y-axis) vs. (A) GM volumes, (B) WM volumes, (C) BMI, or (D) IQ (x-axis), in individuals with PWS (red circles, red solid linear regression line) and controls (blue triangles, blue dashed linear regression line). (For interpretation of the references to color in this figure legend, the reader is referred to the web version of this article.)

For GM volume, there was no significant group x GM volume interaction on brain-PAD, i.e. the slopes of GM versus brain-PAD were not significantly different between controls and PWS groups (*P* = .073, Fig. 3A). Adjusting for group differences in multiple regression analysis, there was an overall negative correlation across the 2 groups between brain-PAD and GM volume (β = −29.17 years [95% CI -47.97, −10.38], SE 9.39, *P* = .003, adjusted r^2^ = 0.25). Adjusting for GM volume, brain-PAD remained significantly greater in PWS than controls (effect size mean ± SEM +6.04 ± 2.09 years [95% CI 1.86, 10.22], *t* = 2.90, *P* = .005) (Fig. 3A).

For WM volume, there was no significant group x WM volume interaction on brain-PAD (*P* = .32, Fig. 3B). Adjusting for group differences, there was an overall positive correlation between brain-PAD and WM volume (β = +38.04 years [95% CI 11.35, 64.74], SE 13.33, *P* = .006, adjusted r^2^ = 0.24). Adjusting for WM volume, brain-PAD remained significantly greater in PWS than controls (effect size mean ± SEM +7.16 ± 2.07 years [95% CI 3.01, 11.31], *t* = 3.45, *P* = .001) (Fig. 3B).

### 3.6 Relationship of brain-PAD with BMI, IQ, sex, behaviour and medication use

For BMI, there was no significant group x BMI interaction on brain-PAD (*P* = .54) (Fig. 3C). There was no significant correlation between BMI and brain-PAD within either group separately (PWS: r = +0.22, *P* = .35; controls r = +0.28, *P* = .085). When combining the groups, adjusting for group differences, there was a trend for an overall positive correlation between brain-PAD and BMI (β = +0.38 years [95% CI -0.02, 0.78], SE 0.20, *P* = .065, adjusted r^2^ = 0.18). However, when adjusting for differences in BMI between groups, by including BMI in the model, brain-PAD remained significantly greater in the PWS compared to control group (effect size mean ± SEM +4.98 ± 2.46 years [95% CI 0.04, 9.91], *t* = 2.02, *P* = .048) (Fig. 3C).

Furthermore, brain-PAD also remained significantly greater in the PWS compared to control group (effect size mean ± SEM +5.51 ± 2.56 years [95% CI 3.44, 10.38], *t* = 2.15 *P* = .037), when matching the groups for BMI, by including only those subjects with PWS having BMI <34.2 kg/m^2^, the maximum BMI in the control group (*n* = 15, BMI median 26.7 [IQR 24.0, 30.0], range 21.5–33.7, 80.0% male), and controls with BMI >21.5 kg/m^2^, the minimum BMI in the PWS group (*n* = 29, BMI median 24.6 [23.3, 27.1], 21.7–34.2, 62.1% male; BMI PWS vs. control: Z -1.02, *P* = .31).

For IQ, there was with no significant group x IQ interaction on brain-PAD (*P* = .12) (Fig. 3D). There was no significant correlation between IQ score and brain-PAD within either group separately (PWS: r = +0.22, *P* = .35; controls *r* = −0.20, *P* = .22), or when combining groups with adjustment for group differences (β = −0.03 years [95% CI -0.21, 0.16], SE 0.09, *P* = .79, adjusted r^2^ = 0.13) (Fig. 3D).

There was no significant sex difference in brain-PAD in either group (PWS: male effect size mean ± SEM -6.63 ± 4.31 years [95% CI -15.68, 2.42], *t* = −1.54, *P* = .14; controls: male effect size 3.11 ± 2.44 years [95% CI -1.83, 8.05], *t* = 1.28, *P* = .21), or in the whole cohort, adjusting for group differences (male effect size −0.04 ± 2.22 years [95% CI -4.48, 4.41], *t* = −0.02, *P* = .99, adjusted r^2^ = 0.13).

In the PWS group, there was no significant correlation between brain-PAD and either repetitive behaviour using RBS-R sameness score (r = +0.26, *P* = .26), or disruptive behaviour using DPC-A disruptive score (r = +0.21, P = .21).

In the PWS group, there was also no significant effect on brain-PAD of medication use, for growth hormone (past or current use: *t* = 0.81, *P* = .43; current use: *t* = 0.57, *P* = .58) or sex hormones (past or current use: *t* = −0.47, *P* = .65; current use: *t* = 0.17, *P* = .87), or current use of any psychoactive medication (*t* = 0.42, *P* = .68), anti-psychotics (*t* = 0.20, *P* = .84) or anti-depressants (*t* = −0.34, *P* = .74).

### 3.7 SNORD116 microdeletion is associated with high brain-PAD score

**Fig. 4:**
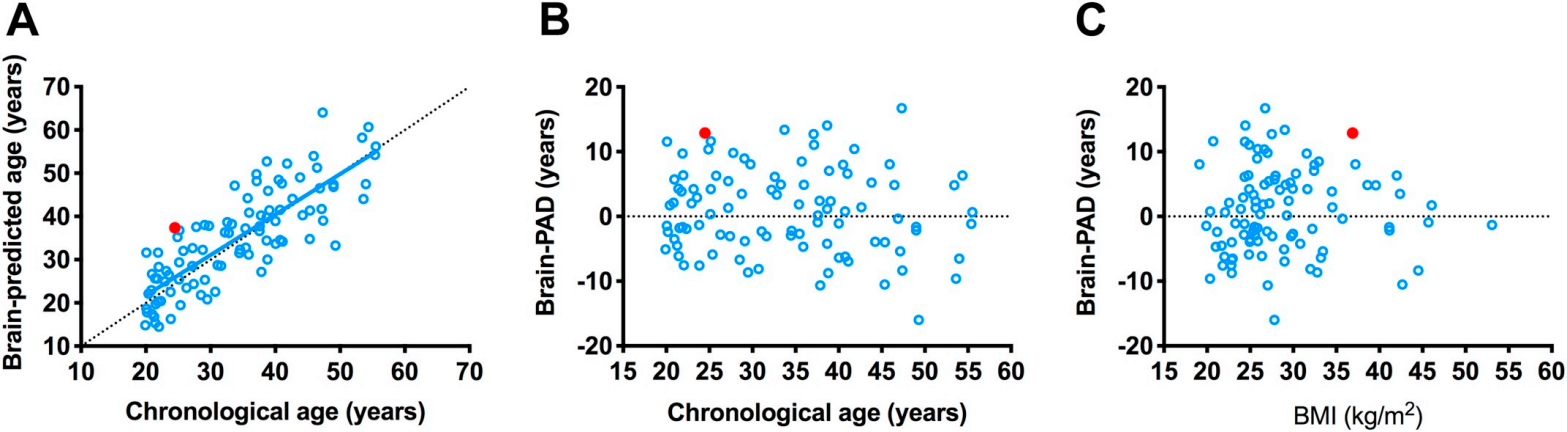
Brain-age prediction in man with SNORD116 microdeletion and controls (cohort 2). Male with a SNORD116 micodeletion (red filled circle) shows an increase in (A) brain age, and (B,C) brain-PAD scores when adjusting for (B) chronological real age or (C) BMI, compared to the control group (unfilled blue circles, and in (A) solid blue regression line). Black dotted line is line of equality (For interpretation of the references to color in this figure legend, the reader is referred to the web version of this article.).

The man with PWS due to a SNORD116 microdeletion had a brain age of 37.36 years compared to an actual chronological age of 24.49 years (Fig. 4A). The resulting brain-PAD score for this patient was +12.87 years, appeared markedly higher than the average brain-PAD of the respective control group (mean ± SD +0.84 ± 6.48 years), including when adjusting for chronological age (Fig. 4B) or BMI (Fig. 4C).

## 4 Discussion

Individuals with PWS had a brain age that more closely resembled healthy adults on average 8.74 years older than their chronological age. This effect was evident relative to age and scanner-matched typically developing controls, in whom no such increase was observed. The findings were independent from lifetime or current receipt of growth hormone or sex hormones, and current use of psychotropic medications. Increased brain age in PWS was also independent of WM and GM tissue volumes, which were not significantly different between groups, and was also independent of the higher BMI in the PWS than control group.

85% of the brain-PAD scores in the PWS scores were positive (17/20), which means that most of PWS individuals in our study showed an increased predicted brain age. However, any variability in brain-PAD scores in the PWS group could not be explained by medication use, including growth or sex hormones or psychotropic drugs, sex or current BMI. In addition to normal individual variation, potential factors might be duration or timing of hormonal replacement, duration or dosage of psychotropic medication, age of onset and duration of obesity, or metabolic complications of obesity. Greater brain age has been associated with poor health outcomes, so the variability might become more relevant with longitudinal follow-up.

Participants with PWS showed signs of premature brain aging and early onset atrophy rather than a fundamental arrest of brain development. Patterns of brain structure more closely resembled healthy older brains rather than healthy younger brains, which might be indicative of premature neuronal loss and atrophy. Even slight decrease in brain tissue volumes might be picked up by the trained model as a biomarker of aging as it has already been established that there is a widespread reduction of GM and WM volumes from young adulthood and onwards (Giorgio et al., 2010).

Previous studies using food intake or food cue related paradigms have revealed functional neuroimaging abnormalities in PWS, focusing mainly on subcortical and cortical structures usually involved in eating behaviour, reward and motivation (Manning and Holland, 2015). However, reports of structural brain abnormalities in PWS have included: ventriculomegaly and cortical abnormalities such as atrophy or abnormal gyrification (Hayashi et al., 1992; Hashimoto et al., 1998; Yoshii et al., 2002; Miller et al., 2007), reduction in total brain volumes or brainstem volume (Miller et al., 2009; Ogura et al., 2011; Lukoshe et al., 2013); and irregularity or malformation of the thalamus, pituitary gland, corpus callosum, insula and sylvian fissure (Leonard et al., 1993; Iughetti et al., 2008; Gilboa and Gross-Tsur, 2013). In the current study, 90% of PWS participants had a normal T1-weighted scan, 1/20 participants had ventriculomegaly and 1/20 participants had arrested hydrocephalus and possible aqueduct stenosis. However, we did not detect any of the other abnormalities reported above, and so were able to distinguish such structural abnormalities from premature brain aging using a quantitative assessment of brain development rather than multiple measures of independent structures and categorization. Analysing widespread structural differences in a cross-sectional population-based study enhances the generalizability of the findings in comparison to the case reports of neuroimaging abnormalities in PWS while studying the brain as a whole.

Interestingly, the higher brain age in PWS was not related to changes in GM and WM volumes which did not differ between controls and PWS groups, emphasizing the strengths of brain age over standard structural volumetrics, as the patterns picked up by the model are spatially distributed. Indeed, more localised examination of structural brain differences in PWS using voxel-based morphometry from the same cohort as in this study, has revealed increased volume in the prefrontal cortex, especially medially, the majority of the cingulate cortices, insula cortices, and areas of the parietal and temporal cortices, caudate, putamen and thalamus, with increased cortical volume largely driven by greater cortical thickness. Reduced volume was found in ventromedial prefrontal areas, medial temporal lobe, bilateral temporal poles, and right lateral prefrontal cortex, while myelination of the cortex was broadly unchanged, with the exception of some highly localised areas, including the insula (Manning et al., 2018).

Our current findings contrast with a previous study reporting that children with PWS showed a fundamentally arrested brain development (Lukoshe et al., 2013). This was especially seen in young children with a chromosome 15q11–13 deletion as compared to children with the maternal UPD genotype. The current study consisted predominantly of patients with the deletion genotype, but did not rely on simply morphological measures determined from the Freesurfer software as in the previous study, but used a whole-brain method based on reference to a model of healthy brain aging defined in an independent sample. Furthermore, our cohort consisted of young adults with PWS rather than children. These differences in methodology and demographics might explain the contrast of results and possibly reveals changes in structural patterns associated with different stages of neurodevelopment.

Although our study is not the first to examine brain structure in young adults with PWS, it is the first to use a pre-defined model of the structural pattern of the brain in healthy adults to compare structural abnormalities in PWS. Moreover, our analysis has an emphasis on markers of aging, known to be associated with a higher risk of death, poorer cognitive function and impaired physical fitness (Cole et al., 2018). The use of a large training set and a well-validated model further confirm the accuracy of the result and draws a clear difference between typical aging and aging in PWS.

It is important to acknowledge the limitations and strengths of our techniques. First and foremost, the sample size of our PWS group is relatively small (*n* = 20), but this is usual in studies of PWS because of the rarity of the disease and the difficulty of recruitment and scanning. Scans from different sources were used for the large training set, for which detailed demographic data were not available. However, it was verified that individuals were free from neurological, psychiatric or mental diseases before using the scans to train the model, and the large number of data used (*n* = 2001) puts the brain-PAD scores of the PWS group in appropriate context. The limited age range of our cohort (both PWS and controls) is both a strength and a constraint, since it eliminates the confounding effect of age, but it may restrict generalizations of the results to other age groups. Therefore, there is still a possibility that brain-PAD scores in subjects with PWS under 18 years of age might be reduced.

In addition, our analysis was cross-sectional, and without further longitudinal studies, the relationship between brain-predicted age, neurological phenotypes and complications, or mortality risk, in association with age, cannot be determined, especially in a cohort with a mean age of 23 years. Cross-sectional studies can only suggest premature brain aging. Given that the control group was recruited at the University of Cambridge, participants might have been exposed to positive influences on apparent brain age, which is further emphasized by the above-average IQ test scores in the control group.

Brain development in utero might already be impaired in PWS patients. Phase 0 of the 4 nutritional phases occurs in utero, during which the foetus shows signs of decreased movements (prenatal hypotonia) and growth restriction (Cassidy et al., 2012; Miller et al., 2011). Moreover, some effects of the loss of function of genes involved in PWS may also be expressed prenatally e.g. they may be confined to the placenta (Robinson et al., 1997). The timing and the nature of birth also seems to influence development in PWS. The position of the foetus at the onset of labor is often abnormal, and a high percentage of children are born either pre- or post-maturely (Swaab, 1997). Premature birth impacts brain structure and development in general, with loss of the neurogenesis stage which is usually still present throughout the third trimester (Malik et al., 2013). Newborns with PWS have a lower BMI than healthy siblings by 15–20% (Miller et al., 2011), indicating early, prenatal, abnormal development. The failure-to-thrive at younger age seems to begin in utero, given that the imprinted genes in PWS are already absent prenatally (Butler et al., 2009). Early work on imprinted genes showed their importance on growth in general, and on neurodevelopment, brain size and organization (Wilkinson et al., 2007). The largest proportion of the discovered imprinted genes in the PWS chromosomal region are expressed, though not exclusively, in the brain, and are key in early brain development. Findings of increased cortical thickness and reduced gyrification in PWS are consistent with disturbances in neuronal migration during foetal cortical development (Lukoshe et al., 2013; Manning et al., 2018).

The complex genetic background of PWS, together with the complicated role of the genes involved in the syndrome might explain some the neurodevelopmental deficits driving our results, as well as the large variability in brain-PAD scores. The exact function and relative importance of each of the genes involved in PWS still needs to be further elucidated, although it is clear that some rare genes are key to the development of the central nervous system in early stages, or in later stages, thus promoting early atrophy.

As an example, some studies have shown that several paternally expressed genes found in the PWS region of mice models are necessary for cortical development. One of the genes, *Necdin (Ndn)*, is a neuronal cell cycle regulator, usually upregulated during development, differentiation and migration of neuronal cells. *Ndn* mutant mice have abnormal neuronal migration and cortical development (Pagliardini et al., 2005). Another gene whose expression is lost in PWS is *Magel2*, important for pre- and post-natal development specifically of the hypothalamus (Lee et al., 2003). The deficits associated with the loss of function of *Magel2* might worsen with age, due to the lack of clearance of ubiquitinated proteins from the cells (Pravdivyi et al., 2015), which could contribute to neuroinflammation and activation of stress pathways. A recent report of gene expression in post-mortem hypothalamic tissue in PWS reported a transcriptomic profile similar to that seen in neurodegenerative conditions and the aging brain, with downregulated genes mainly found in neuronal cells and involved in neurogenesis and synaptic plasticity, while upregulated genes were mainly microglial and associated with inflammatory responses (Bochukova et al., 2018). In addition, a recent small study found decreased ex vivo mitochondrial function in fibroblast cell lines from patients with PWS (especially in those with deletion genotype) that might also play a pathogenic role in accelerated brain aging if also seen in other cell types (Butler et al., 2018).

Study of rare mutations or microdeletions of specific genes can also reveal much about genotype-phenotype association. A strength of the brain age study design is that individualised predictions can be made, allowing comparison of single subjects to a model of normal brain aging. We also examined the brain-PAD score of a 24.5 years old male with a genetically confirmed 187 kb microdeletion at chromosome 15q11–13 (de Smith et al., 2009). He had an advanced brain-PAD of +12.87 years, compared to a separate set of control adults (that appeared independent of chronological age and BMI). This patient displays hyperphagia, obesity, hypogonadism and other features commonly associated with PWS. This microdeletion encompasses a family of paternally expressed, maternally imprinted, non-coding RNAs, particularly the *SNORD116* small nucleolar RNA (snoRNA) cluster. Given that this patient exhibits a similar increase in brain-PAD score within the range of brain-PAD scores of our PWS cohort, we hypothesize that the loss of function for this genetic cluster might be sufficient to cause premature brain aging. Two studies on mice lacking the *snord116* gene cluster showed growth and motor learning deficiencies, and a possible hyperphagic phenotype (Ding et al., 2008; Qi et al., 2016), although more work is needed to understand the impact of the snoRNAs on brain structure and development.

Some genotype-phenotypes associations between different genetic subtypes resulting in PWS, have been described, such as differences in autistic features, risk of psychosis in adulthood, and differential brain development (Mitchell et al., 1996; Boer et al., 2002; Soni et al., 2008; Sinnema et al., 2011; Lukoshe et al., 2013). It should be noted that the vast majority of subjects in the current study had a paternal chromosome 15q11–13 deletion, with only 1 out of 20 having mUPD. However, this subject with mUPD had a brain-age score well within the range of the PWS group with a deletion.

Aside from the varying genetic background that might be a cause for the atypical brain development, PWS is also the most common genetic syndromal cause of morbid obesity. Evidence points at an increase in oxidative stress associated with both obesity and aging (Furukawa et al., 2004), with a higher production of pro-inflammatory cytokines interacting with the brain (Chung et al., 2009; Arnoldussen et al., 2014). Interleukin-6 and tumor-necrosis factor, found in both the periphery (produced by fat cells) and the brain (produced by neurons, astrocytes and microglia), which normally contribute to neurogenesis, neuroinflammation and synaptic plasticity, seem to be linked to cognitive decline, neurodegeneration and brain atrophy in obese people (Wilson et al., 2002; Griffin, 2006), as well as increased risk of dementia and Alzheimer's disease (Gustafson et al., 2004; Whitmer et al., 2005). Moreover, leptin, a hormone produced by adipose tissue, seems to be related to inflammatory response in microglia, with a positive feedback loop for sustained cytokine production, which are in turn associated with WM changes (Wisse, 2004; Bolzenius et al., 2013; Kullmann et al., 2015). However, there is no evidence of different leptin concentrations in the serum of PWS individuals (Goldstone et al., 2002), but evidence suggests activation of the innate immune system independently of hormonal imbalance in obesity (Viardot et al., 2010). In PWS, all of these factors might lead to differential brain development and aging depending on the age and the genetic predisposition of the patients.

Previous structural neuroimaging studies have shown that BMI is associated with decreased volume of the limbic and frontal GM regions in healthy children (Alosco et al., 2014) and widespread brain regions in adults (Cole et al., 2013). These changes are in turn related to cognitive decline and other neurocognitive outcomes (Gunstad et al., 2008). A recent study also showed that WM volume in overweight and obese people was associated with premature cortical atrophy and an increased brain age of 10 years from middle age, with differential effects depending on brain tissue types (Ronan et al., 2016). There is also evidence that BMI is associated with global decrease of WM microstructural integrity (Verstynen et al., 2012). In the current study, there was an overall trend across combined control and PWS groups for higher BMI to be associated with a higher brain-PAD, with each 1 kg/m2 increase in BMI advancing brain-PAD by +0.38 years. However, the higher brain-PAD in PWS than controls was not explained by the higher BMI in PWS, since it persisted when adjusting for BMI, and when restricting analysis to a sub-cohort matched for BMI. It remains possible that with even more extreme obesity in PWS that there is an interaction between the effects of PWS, obesity and its metabolic complications, on accelerating brain aging. Unfortunately, no questionnaire or behavioural measurements of hyperphagia were available in the PWS group, to examine any bidirectional association between brain aging and overeating behaviour in PWS, independent of BMI. This would be useful to examine in future studies. Furthermore, the small sample size may have caused a type 2 error in any influence of sex on brain-PAD, as there was a trend for brain-PAD to be lower in men than women with PWS, though the PWS group was also predominantly male.

In future investigations of PWS, the use of a larger age range, especially including children and adolescents, individuals with more severe obesity, and a longitudinal study would help uncover the natural history of brain development and aging and the interaction with development of obesity. Understanding how brain aging differs between the different genotypes (especially mUPD (or imprinting centre microdeletions and imprinting defects) vs. paternal deletion, type I vs type II deletions, and rarer microdeletions involving snoRNA clusters, and specific gene mutations e.g. *MAGEL2*, *MKRN3* genes) would also help understand the role of specific genes in the PWS regions, potential mechanisms leading to premature aging in PWS, role for gene dosage outside of the PWS critical region, and the potential neurobiological basis of genotype-phenotype interactions in PWS, for example the increased risk of psychosis with mUPD. Given the widespread neurological effects of PWS, it will be especially helpful to incorporate different imaging and analysis techniques such as DTI and structural and functional MRI and multi-modality brain-age prediction in the study of older patients with PWS.

Further understanding of the potential role of obesity and associated metabolic factors, and PWS genotypes, in the development of increased brain aging in PWS will emphasize additional reasons to develop strategies and novel therapies to prevent and treat overeating, weight gain and metabolic syndrome, and perhaps even targeted to particular genotypes. Furthermore, as adults live longer with PWS with earlier diagnosis and improved multi-disciplinary care, including the use of specialist residential placements, the presence of accelerated brain aging identified through MRI brain scans, may identify individuals at risk of cognitive decline in later adulthood, perhaps through neurodegenerative pathological processes leading to dementia, who may need closer monitoring, aggressive treatment of reversible risk factors and perhaps early use of pharmacological therapies (Sinnema et al., 2010; Sinnema et al., 2012; Whittington et al., 2015).

In conclusion, this neuroimaging study indicates that PWS is associated with an increased brain-age from early adulthood. This conclusion challenges the competing hypotheses (arrested development vs. premature aging) trying to explain structural brain abnormalities in PWS. This approach suggests that there may be neurological implications of the syndrome in older age, which may have important clinical implications as the life expectancy of patients with PWS is prolonged with better management of the food environment and avoidance of obesity-related complications.

## Appendix A Supplementary data

Supplementary Table 1. Data sources for healthy brain age training sample. Image 1
